# Syphilitic Hepatitis: A Rare Cause of Acute Liver Injury

**DOI:** 10.7759/cureus.14800

**Published:** 2021-05-02

**Authors:** Ahmed Alemam, Subhan Ata, Danial Shaikh, Bianca Leuzzi, Jasbir Makker

**Affiliations:** 1 Gastroenterology, BronxCare Health System, Bronx, USA; 2 Internal Medicine, BronxCare Health System, Bronx, USA; 3 Medicine/Gastroenterology, BronxCare Health System, Bronx, USA; 4 Gastroenterology, American University of the Caribbean, Cupecoy, SXM

**Keywords:** syphilis, liver injury, hepatitis, transaminitis

## Abstract

Syphilitic hepatitis represents a rare manifestation of treponemal infection. Diagnosis is achieved with the presence of characteristic syphilitic signs and symptoms along with positive serological markers, characteristic elevated liver enzymes, and no other alternative cause of hepatobiliary insult. Here we detail a case of a patient presenting with recently diagnosed secondary syphilis causing abnormal liver enzymes. With the increasing incidence of venereal diseases in the United States, this case highlights the importance of identifying syphilis as a differential diagnosis for acute liver injury.

## Introduction

Syphilis is a disease caused by the bacterium *Treponema pallidum*. This disease is usually associated with high-risk sexual activity, especially among men who account for 86% of syphilis cases [1]. Syphilis will progress from primary to secondary and eventually to the tertiary stage if left untreated. Primary syphilis usually involves a single painless ulcer that spontaneously resolves. Secondary syphilis presents with systemic symptoms weeks to months after the initial inoculation, with a rash most commonly involving the hands and feet. Tertiary syphilis occurs years after initial infection and can present with devastating neurological and cardiac manifestations [1]. Since 2001, primary and secondary syphilis are on a rise in incidence year after year [2]. During 2018, a total of 35,063 cases of primary and secondary syphilis were reported in the United States, yielding a rate of 10.8 cases per 100,000 population [2]. Here we present a rare case of syphilitic hepatitis.

## Case presentation

A 42-year-old African American male with a past medical history of gout, hypertension, and recurrent genital herpes presented to our Emergency Department complaining of right upper quadrant and epigastric abdominal pain with nausea and non-bilious, non-bloody vomiting of one-day duration. Epigastric pain occurred after eating a meal consisting of lobster and steak. It was described as a cramping, intermittent, non-radiating pain with moderate severity and no aggravating or alleviating factors. He denied any fever, chills, diarrhea, hematochezia, yellowing of skin, genital lesions, or burning micturition. He had no recent travel or sick contacts. He was started on allopurinol (100 mg, once a day) 10 days before presentation for longstanding gout and was taking valacyclovir (500 mg, once a day) for recurrent genital herpes. Detailed medication history also revealed the use of herbal medication (black seed bitters) around three months prior to presentation. He reported occasional marijuana use but denied tobacco consumption, excessive alcohol consumption, acetaminophen use, or any other recreational drug use. He denied any family history of hepatobiliary disease. He had a surgical history of anterior cervical discectomy and fusion (ACDF) for a cervical herniation secondary to car accident two years prior to presentation.

**Figure 1:**
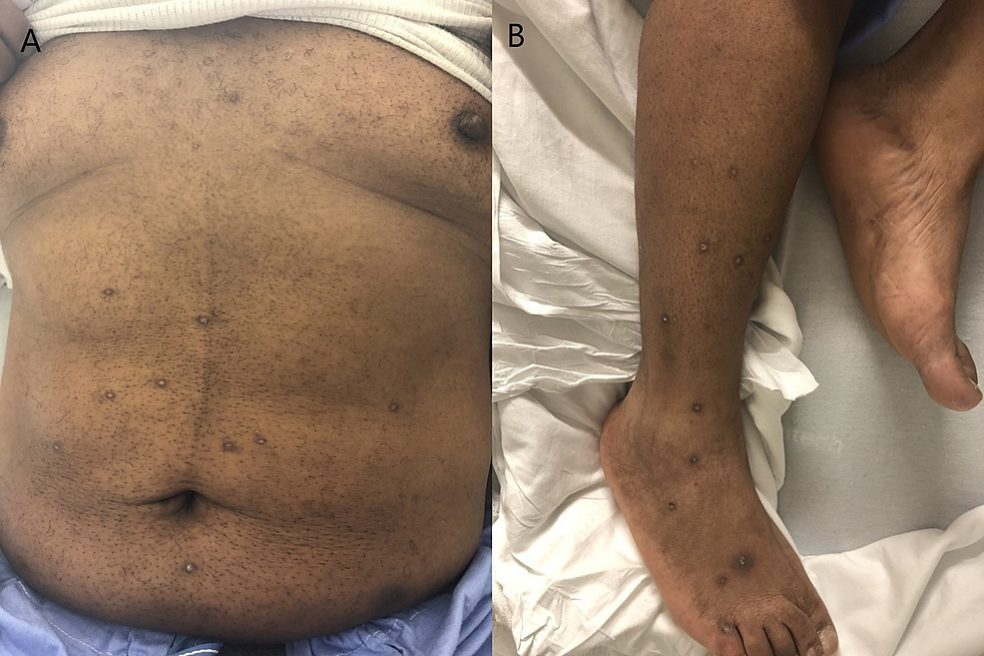
Patient with hyperkeratotic papular rash on the torso (a) and legs (b)

Vital signs on presentation showed blood pressure of 126/68 mm Hg, pulse of 73 beats per minute, temperature of 99.1°C, and respiratory rate of 15 breaths per minute with oxygen saturation of 100% on room air. Physical examination revealed age-appropriate skin changes and no acute distress. He had a horizontal occipital scar from a remote injury in his childhood and lateral neck scar from ACDF. He had scattered hyperkeratotic papular rash on the arms, torso, and legs (Figure 1), and herpetic lesions on the glans penis. Cardiopulmonary examination revealed findings within normal limits. Abdominal examination showed tender epigastrium with rest of the abdomen soft, non-tender, with no masses or organomegaly. Neurological examination was grossly within normal limits.

Initial investigations were remarkable for cholestatic liver injury with serum alkaline phosphatase (ALP) at 659 IU/L (normal: 52-128 IU/L), aspartate transaminase (AST) at 103 IU/L (normal: 9-48 IU/L), alanine transaminase (ALT) at 152 IU/L (normal: 5-40 IU/L), gamma glutamyl transferase (GGT) at 802 IU/L (normal: 8-54 IU/L), albumin at 3.3 g/dL (normal: 3.4-4.8 g/dL), total bilirubin at 0.5 mg/dL (normal: 0.2-1.1 mg/dL), and direct bilirubin at 0.3 mg/dL (normal: 0.0-0.3 mg/dL). Ultrasound of the abdomen demonstrated increased liver echogenicity without hepatomegaly, normal gallbladder, non-dilated common bile duct, normal pancreas, and patent portal hepatic flow.

The patient was admitted to the hospital for further investigation of hepatic injury. At the time, differential diagnosis included possible drug-induced liver injury (DILI) secondary to allopurinol, autoimmune hepatitis, or biliary disease. Allopurinol was discontinued due to concern for DILI and drug reaction with eosinophilia and systemic symptoms syndrome (DRESS). Viral hepatitis serology demonstrated hepatitis A total antibody positive, hepatitis C antibody negative, hepatitis B surface antibody positive, and herpes simplex virus (HSV) IgM polymerase chain reaction (PCR) negative. Autoimmune liver profile demonstrated normal immunoglobulin G levels and absence of anti-nuclear, liver kidney microsomal, smooth muscle, and anti-mitochondrial antibodies. Acetaminophen levels were <14.9 ug/mL, below normal range for toxicity.

On further questioning, the patient revealed that one week before presentation, he was diagnosed with syphilis by his primary care physician. His syphilis diagnosis was preceded by a two-month history of polyarthralgia, diffuse pruritus, intermittent right upper quadrant abdominal pain, sore throat, fatigue, “cognac” colored urine, and weight loss. The patient had received one dose of penicillin G benzathine (2.4 million units intramuscularly). His diagnosis was serologically confirmed with reactivity on nontreponemal (RPR titer 1:64) and treponemal testing (IgM fluorescent T pallidum antibody absorbance [FTA‐Abs]). The patient had blood tests done at his primary care provider's office and he was noted to have abnormal liver function tests prior to the admission (ALP of 722 IU/L, AST of 82 IU/L, ALT of 157 IU/L, albumin of 2.7 g/dL) which predated the recent start of allopurinol, and had normal liver function tests with albumin of 4.2 g/dL, AST of 18 IU/L, ALT of 32 IU/L, and ALP of 55 IU/L one year prior. Based on this information, DILI secondary to allopurinol was excluded, and syphilitic hepatitis became our primary working diagnosis.

The patient’s liver function tests were monitored daily, and a steady downward trend was noted towards resolution. He was discharged after a six-day hospital course with last in-hospital ALP of 461 IU/L, AST of 43 IU/L, and ALT of 79 IU/L (Table 1). The patient was instructed to complete syphilis treatment by his primary care physician where he was scheduled to receive his remaining two doses within two weeks. He was advised to follow up in our gastroenterology and hepatology clinic to repeat liver function tests and monitor for resolution of presumed syphilitic hepatitis.

**Table 1:** Liver biochemical profile of our patient before and after antimicrobial treatment AST, aspartate transaminase; ALT, alanine transaminase; GGT, gamma glutamyl transferase

	Baseline (one year ago)	One month prior to hospital admission	Hospital day 0	Hospital day 5
Alkaline phosphatase (normal: 52-128 IU/L)	55	722	659	461
AST (normal: 9-48 IU/L)	18	82	103	43
ALT (normal: 5-40 IU/L)	32	157	152	79
GGT (normal: 8-54 IU/L)	-	-	802	-
Albumin (normal: 3.4-4.8 g/dL)	4.2	2.2	3.3	3.5
Total bilirubin (normal: 0.2-1.1 mg/dL)	-	-	0.5	0.5

## Discussion

Syphilitic hepatitis can be defined as a cholestatic pattern of liver enzyme elevation with serological treponemal evidence in the absence of alternative causes of hepatic dysfunction. The most common stage that causes abnormal liver enzymes is secondary syphilis. It is estimated that 3% of secondary syphilis cases can present as syphilitic hepatitis, but among all the patients with syphilis, hepatitis occurs in 0.2% to 3% of patients [3,4]. A literature review of 97 cases by Huang et al. in 2018 demonstrated that the most common clinical manifestations of syphilitic hepatitis were rash (78%), fatigue/poor appetite (57%), icterus (35%), fever (26%), weight loss (23%), abdominal pain (22%), phallodynia (13.4%), sore throat (8.2%), and headache 7.2%. Physical examination findings demonstrated hepatomegaly (54%) and lymphadenopathy (31%). Liver function tests demonstrated a mean total bilirubin of 8.2 mg/dL, mean ALT of 314.5 U/L, mean AST of 253 U/L, mean ALP of 84.5 U/L, and mean GGT of 561.8 U/L [5]. Although no clear diagnostic criteria exist, Mullick et al. proposed that a reasonable diagnosis without pathology can be elucidated by abnormal liver enzyme levels in a cholestatic pattern, serological evidence for syphilis, exclusion of other causes of liver diseases, and liver enzyme levels returning to normal after appropriate antimicrobial therapy [6]. Our patient demonstrated all four criteria; however, the patient was lost to follow-up before full resolution could be demonstrated. It is postulated that the cholestasis pattern of liver injury seen with syphilitic hepatitis may be due to infection via receptive anal intercourse with subsequent migration to portal circulation and infiltration of bile duct causing pericholangial inflammation [3,7,8]. This is indirectly supported by evidence showing that most syphilitic hepatitis occurs in human immunodeficiency virus (HIV) positive men who have sex with men (MSM) with syphilitic proctitis a precursor in some patients [5,8]. Most cases of syphilitic hepatitis resolve after treatment of syphilis. Fulminant hepatitis is rare, and literature review demonstrates two known cases of syphilis-induced hepatic failure resulting in transplantation and death [9,10]. In our case, the patient had initiated syphilis treatment prior to presentation, and with prior records serving as a baseline (Table 2) we observed a downward trend toward hepatic resolution. The patient was subsequently lost to follow-up.

**Table 2:** Patient’s baseline biochemical values

	Hematocrit	Platelet	Prothrombin	International normalized ratio	Sodium	Potassium	Blood urea nitrogen	Creatinine	Total protein
Initial labs	35.6% (normal: 42-51%)	241 (normal: 150-400 k/uL)	11.2 (normal: 10.7-12.9 seconds)	0.95 (normal 0.90-1.09)	139 (normal: 135-145 mEq/L)	4.5 (normal: 3.5-5.0 mEq/L)	15 (normal: 8-26 mg/dL)	1.2 (normal: 0.5-1.5 mg/dL)	6.6 (normal: 6.0-8.5 g/dL)

## Conclusions

Syphilitic hepatitis is a rare but an important cause of abnormal liver enzymes. Syphilis infection should be a consideration in patients with high-risk features (HIV positive, MSM, and multiple sexual partners) and disproportionate elevation of alkaline phosphate. The importance of identifying syphilis as a cause of elevated liver enzymes lies in the ease of resolution of transaminitis with treatment, thus avoiding unnecessary and expensive workup.
